# Unintended consequences of selection for increased production on the health and welfare of livestock

**DOI:** 10.5194/aab-64-177-2021

**Published:** 2021-05-25

**Authors:** Este van Marle-Köster, Carina Visser

**Affiliations:** Faculty of Natural and Agricultural Sciences, Department of Animal Science, University of Pretoria, Pretoria 0028, South Africa

## Abstract

Modern farming technologies, including quantitative selection and breeding methods in farm animal species, resulted in increased production and efficiency. Selection for increased output in both intensive and extensive production systems has trade-offs and negative outcomes, often more pronounced in intensive systems. Animal welfare and health are often adversely affected and this influences sustainable production. The relative importance of animal welfare differs among developed and developing countries due to the level of economic development, food security and education, as well as religious and cultural practices which presents challenges for sound scientific research. Due to breeding goals in the past set on growth performance, traits such as fertility, welfare and health have been neglected. Fertility is the single most important trait in all livestock species. Reduced fertility and lameness, claw health and mastitis results in unnecessary culling and reduced longevity. Selection pressure for growth accompanied with inbreeding has resulted in a number of genetic defects in beef, sheep and pigs. This review demonstrated the importance of inclusion of animal welfare concepts into breeding objectives and selection strategies. Accurate phenotyping of welfare traits is a limiting factor in the implementation of mitigating strategies, which include diagnostic testing, control of inbreeding and genomic selection.

## 1 Introduction

The selection of farm animals commenced with the domestication of the various livestock species approximately 12 000 years ago, with the primary aim to benefit humankind in providing food, hides and fibres (Driscoll et al., 2009). A large variety of modern domesticated dairy and beef cattle, goat, sheep and pig breeds originated from their respective ancestors, which included the auroch (*Bos primigenius*), bezoar (*Capra hircus*), mouflon (*Ovis aries*) and wild boar (*Sus scrofa*) (Diamond, 2002).

Over many centuries, livestock, pigs and poultry were farmed under a wide range of environmental conditions in different parts of the world. The arrival of various technologies resulted in modern production systems with advanced housing, feeding and selection and breeding methodologies for farm animal species that resulted in higher production output and increased efficiency. However, biological and scientific advances, such as selection for increased production, often have trade-offs and negative outcomes within the system.

Quantitative selection practises have dominated the formulation of breeding objectives and selection over the past century. The development of animal recording systems resulted in a data-driven approach to formulation of breeding objectives. Garrick and Golden (2008) emphasised the potential dangers of using only traits that are easily recorded in selection programmes. These traits, such as growth traits, have moderate to high heritability estimates and thus result in relatively fast genetic progress. This approach has however neglected the traits not easily recorded or costly to record, including traits associated with health and animal welfare. Some of the deleterious consequences in livestock production came about via direct selection, while others were unintended. The adverse effects on health and welfare traits have generally been more pronounced in intensive production systems, such as lameness in dairy cattle and pigs.

It is important to take note of the change in the interaction between humans and their livestock over centuries. In ancient times and through the Middle Ages, humans lived in a close relationship with their animals. Many communities throughout the world were dependant on some form of agriculture, and the majority of agricultural production was based on family farming. In developed countries, over many centuries, various factors contributed to a change in livestock farming, away from traditional family farms to larger commercial production units with changed labour and management structures (Lowder et al., 2016). This has arguably also resulted in less close contact between farmers and their animals. In developing countries (as in Africa), a substantial number of livestock are kept in communal and smallholder systems where livestock serve several more purposes than only being a food source (Mapiye et al., 2019). In these systems, different challenges are faced with regard to animal health and welfare, such as the common practise of tethering (Mataveia et al., 2018).

Selection of livestock in both intensive and extensive production systems is under pressure to meet the increased demand for animal protein by a growing world population which is estimated to reach 9 billion humans by 2050 (Godfray et al., 2010; Telugu et al., 2017). Furthermore, selection programmes should address the reduction of the carbon footprint and methane emissions and adhere to the sustainable development goals (SDGs). The consumer has also become more conscious with regard to the origin of food production, standards and safety regulations. All these aspects are important in the formulation of sustainable breeding objectives, and specific attention should be given to traits related to animal welfare. The consumer has entered the debate on animal welfare, often accompanied by emotion and little scientific reasoning (Buller et al., 2018). It is therefore the responsibility of the animal science community to recognise welfare traits as important to the sustainability of the respective industries.

In this review, we firstly provide context on animal welfare with regard to the current world perspective, followed by a discussion of the unintended consequences of selection on welfare and health traits with reference to extensive and intensive production systems. Mitigating strategies are reviewed with specific reference to welfare traits.

## 2 Defining welfare in livestock

In a broad sense, the word “welfare” is defined as the state in which an animal or human is content, safe and healthy (Oxford University, 2009). The first definitions of animal welfare referred to the complete mental and physical health of the animal in harmony with its environment as well as free from suffering (Hughes, 1976; Carpenter, 1980). More recent definitions state that the welfare of any sentient farmed animal, or animal used in the service of human beings, should be defined by the animal's individual perception of its own physical and emotional state (Webster, 2013). The majority of these all refer to physical health, environment and the emotional and psychological state of the animal (Koknaroglu and Akunal, 2013). It could be argued that in the case of farm animals, their physical health and production environment tend to be the primary focus with regard to animal welfare, as these aspects will have an influence on the production outcomes and the profitability of the system. Animal welfare should therefore be considered in selection to avoid genetic defects or outcomes, which will result in unwanted culling, pain or physiological stress. The physiological and behavioural aspects are more difficult to measure objectively and often not considered in selection programmes.

In 1992, the Farm Animal Welfare Council compiled the “Five Freedoms of Animals” in an attempt to improve and regulate the wellbeing of all farm animals (Manteca et al., 2012), which includes freedom from hunger and thirst, freedom from discomfort, freedom from pain, injury or disease, freedom to express normal behaviour and freedom from fear and distress. These freedoms largely pertain to the living conditions and care of an animal. Furthermore, in 2008, the International Committee of the World Organisation for Animal Health (also known by its French acronym: *Office International des Épizooties*, OIE) passed a resolution appealing for affirmation of their standards of animal welfare as the global reference standard for OIE members. Consequently, in 2012, the 178 members of the OIE adopted 10 “General Principles for the Welfare of Animals in Livestock Production Systems” (OIE, 2012), which are summarised in Table 1.

**Table 1:** Principles for the welfare of animals in livestock production (adapted from OIE, 2012).

Broad category	Principle description
Genetic selection	The effect on animal health, behaviour and temperament
External environment	The influence on injuries and the transmission of diseases and parasites
Management	The effect on resting, movement and the performance of natural behaviour of groups to minimise conflict and allow positive social contact
Housing	The effects of air quality, temperature and humidity on animal health and comfort
Nutrition	Ensuring access to feed and water suited to the animals/species needs
Veterinary and healthcare	Prevention and control of diseases and parasites, with humane euthanasia if treatment is not feasible or recovery is unlikely; prevention and management of pain
Emotional	Creation of positive human–animal relationships
Human care andhandling	Ensuring adequate skills and knowledge among animal handlers

The relative importance of animal welfare differs among countries due to a variety of reasons that include the level of economic development and food security, the level of education as well as religious and cultural practises (Wilkens et al., 2005; Sinclair et al., 2019). Perceptions of animal welfare within the scientific community vary, which also presents challenges for sound scientific research and maintaining animal welfare as a scientific field (Korte et al., 2007; Von Keyserlingk and Hötzel, 2015).

Policies and statutory regulations on animal welfare vary among countries. In many developed countries, animals are regarded as sentient creatures with a trend towards increased anthropocentric thinking regarding the treatment of animals (Korte et al., 2007). This may lead to an increased influence of social and cultural opinions on legislation affecting animal production and in effect may have a negative impact on the global improvement of animal welfare.

In Table 2, a list of non-governmental animal welfare organisations around the world is provided. This is not an exhaustive list, but it demonstrates the recognition of animal welfare around the world.

**Table 2:** Examples of non-governmental animal welfare organisations around the world and the years in which they were founded.

Name of the organisation	Year	Reference (last access: 17 September 2020)
	founded	
World Animals Protection (WPSA)	1981	https://www.worldanimalprotection.or.ke/change-wspa
International fund for Animal Welfare (IFAW)	1969	https://www.ifaw.org/
Compassion in World Farming (CIWF)	1967	https://www.ciwf.com
Royal Society for the Prevention of Cruelty to Animals (RSPCA)	1824	https://www.rspca.org.uk/
Eurogroup for Animal Welfare	1980	https://www.eurogroupforanimals.org/
National Council of Societies for the Prevention of Cruelty to Animals (NSPCA) South Africa	1955	https://nspca.co.za/
Japan Farm Animal Welfare Initiative	1973	https://www.esdaw.eu/society-and-animal-welfare-Japan
RSPC Australia	1981	https://www.rspca.org.au/
NSPCA New Zealand	1933	https://www.spca.nz/

These organisations aim to protect a large number of animal species, including farm animals. Besides the non-governmental organisations, there are international animal welfare science organisations with the purpose to cover all aspects of ethology and interactions between humans and animals, including farm animals (Bayvel and Cross, 2010). It is important that animal geneticists and scientists consider animal welfare traits in research efforts to ensure long-term sustainable production outcomes.

## 3 Unintended outcomes of selection

Welfare as mentioned in the section above is complex and constitutes more than the emotional and physical wellbeing of the animal (Koknaroglu and Akunal, 2013). While emotional and physiological welfare traits remain challenging to assess, there are a number of traits, which directly or indirectly may influence the wellbeing and production of the animal, which can be measured and included in selection programmes.

The setting of breeding objectives, and thus selection progress, was historically driven by the economic value of traits. Livestock industries aimed to make the most progress in the production traits that would correspond to the highest profit (Miglior et al., 2017). This however was associated with a decline in other traits, mainly associated with reproduction and welfare. The focus of breeding objectives on production in combination with a data-driven selection approach have resulted in unintended outcomes in a number of traits. In the seed stock industry, especially for dairy and pigs, genetic defects (e.g. CVM: complex vertebral malformation syndrome, BLAD: bovine leukocyte adhesion deficiency, MHS: malignant hypothermia-susceptible) have become more prevalent where unintended inbreeding has been practised with the extensive use of superior sires via artificial insemination (AI). Although a number of countries have made a shift towards a more balanced approach regarding the choice of selection objectives, it will take time to correct the adverse effects of the previous strategy.

The relationship between welfare and reproduction traits is complex and influenced by many factors. Although the relationship between welfare and fertility is not linear (i.e. some animals can maintain high levels of productivity in the short term while being compromised in terms of welfare aspects), a decrease in reproductive efficiency is associated with increased stress (Ritter et al., 2019). The decline in fertility results in an increase in involuntary culling of the animals; therefore, it is a reasonable inference that fertility can be accepted as a welfare trait.

Fertility is considered the single most important economic trait in both intensive and extensive production systems (Cammack et al., 2009; Walsh et al., 2011). Heritability estimates for fertility traits are generally low due to the high environmental variance. Intensive selection for production traits in the past has unintentionally led to selection for impaired fertility, due to the unfavourable genetic correlations between these components. This decline in fertility is the most pronounced in dairy cattle where milk yield, fat yield and protein yield have been the major focus (Berry et al., 2014). In species such as dairy cattle, a vast amount of research is available on the adverse effects of directional selection for milk yield on cow fertility, health and welfare (Berry et al., 2003; Dillon et al., 2006; Roche, 2006; Zink et al., 2011; Berry et al., 2014; Berry and Evans, 2014). In the dairy industry, the use of AI has contributed to inbreeding, resulting in a decline in overall fertility (Weigel, 2001). Genetic defects such as bovine leukocyte adhesion deficiency (BLAD), deficiency of uridine monophosphate synthase (DUMPS) and complex vertebral malformation (CVM) are related to the overuse of specific superior sires and have been well documented (Cole, 2015).

Dairy cow fertility is linked to cow lameness, which is also related to claw health. Lameness is associated with pain and stress, and the stress response is thought to have a negative influence on the reproductive cycle, causing a decrease in number of eggs ovulated, as well as a decrease in fertilisation rate (Fitzgerald et al., 2012). Lameness is associated with claw lesions (including non-infectious and infections disorders) which are mostly observed in cows housed in intensive production systems requiring higher management inputs. Claw defects such as cork screw claw, dermatitis, heel horn erosion, sole ulcers and white line disorder have been reported to have a genetic basis (Heringstad et al., 2018) and can be addressed in selection if accurately recorded on farm. Solutions for improved claw welfare will require a multifactorial approach.

Although health traits are beyond the scope of this review, mastitis must be mentioned as an important welfare trait. This is a complex trait in terms of recording and it is influenced by a range of physiological and management factors. Selection for resistance to mastitis should result in resistance to other diseases, as well as improved reproduction efficiency and longer herd life of dairy cows (Martin et al., 2018).

In pig production systems, reproductive failures contribute to over 50 % of involuntary culling after the first farrowing (Rauw, 1999). The use of AI in pig production systems has resulted in increased inbreeding rates, potentially causing inbreeding depression as well as increasing the frequency of deleterious alleles (Andersson, 2013). These mutations are usually present at low frequencies, with the alleles widespread throughout populations (Leroy, 2014). Intense selection and the use of AI have resulted in decreased effective population sizes that led to increased frequencies of these deleterious alleles, resulting in increased prevalence of embryonic death caused by lethal mutations. This also affects fertility, lowering the reproductive performance in pig populations (Derks et al., 2019).

Lameness is a recognised problem in pigs (Le et al., 2015; Nalon and Stevenson, 2019) and affects pig production on both a welfare and economic level. Le et al. (2015) reported that boars with leg deformities and lameness showed a decreased daily weight gain, resulting in decreased profitability and efficiency. Sows with superior moving ability were likely to show superior fertility, with larger litter sizes and shorter between-parity intervals. Nalon and Stevenson (2019) added that locomotory disorders were the major cause of involuntary culling of healthy breeding sows.

Lameness has been also associated with claw quality in beef cattle. Heritability estimates for claw traits remain low due to large environmental variances, and improved objective measurements are required for genetic evaluations. In a study on Angus cattle in the US, heritability estimates varied from 0.16 to 0.37 for different claw traits (Wang et al., 2017). In a South African study on Bonsmara cattle, the morphological and physiological characteristics of claws were studied, indicating that tensile strength was influenced by bioregion. It was also noted that the differences observed between front and hind claws could be related to selection pressure on conformation and accelerated growth (Van Marle-Köster et al., 2019).

Beef and sheep are raised mostly in extensive farming systems with breeding objectives biased towards growth traits. Webb and Casey (2010) have reviewed the consequences of prolonged selection for growth and growth efficiency on meat quality in detail. Selection emphasis on increased muscularity and meat yield in beef cattle has inadvertently led to conditions such as double muscling with adverse effects on fertility (Fiems, 2012; Fiems and Ampe, 2014). The inactivation of the myostatin (*MSTN*) gene causes an increase in skeletal muscle weight, but at the same time, fertility decreases with an increase in dystocia and a reduction in calf survival (Arthur, 2004; Greger, 2011). A number of myostatin variants have been identified in beef cattle breeds using genomics, and these are a reason for concern with regard to animal welfare.

Selection for accelerated growth also posed welfare problems in sheep and goat breeding. The callipyge gene in sheep has been reported to cause a similar double-muscled phenotype in sheep to that seen in beef cattle; however, a study done by Jackson et al. (1997) indicated that none of the adverse effects associated with double muscling in beef cattle are seen in callipyge sheep. A decrease in terms of meat tenderness have however been reported (Webb and Casey, 2010).

In pigs, increased occurrences of metabolic diseases such as porcine stress syndrome (PSS) and mulberry heart disease have been linked to the genetic selection for increasingly fast growing, lean animals, with large muscle blocks (Brambilla et al., 2002). PSS is a major contributor to the occurrence of PSE (pale, soft and exudative meat) and has been linked to a recessive mutation on chromosome 6, characterised as a single-nucleotide substitution (T/C) in the gene coding for porcine calcium release, also referred to as the skeletal ryanodine receptor 1 gene (*RYR1* locus) (Ile et al., 2018). A number of other muscle defects (such as dark firm dry (DFD) syndrome) have been reported in pigs (Webb and Casey, 2010).

Few welfare studies on sheep and goats are available, which is largely attributed to the predominantly extensive systems in which they are produced (Sevi et al., 2009). Both the ability of small stock to adapt to severe environments and their robustness are often exaggerated, leading to perceptions that inadequate management practises and unfavourable environments do not have large influences on their health and welfare (Sevi et al., 2009). Husbandry practices that do impact adversely on sheep include surgical mulesing and tail docking. Mulesing is the removal of skin around the breech area and is generally regarded as a painful procedure, as is tail docking (Small et al., 2018). Mulesing has been banned in some countries (i.e. New Zealand) but is still legal in many others, including developed countries such as Australia. There is a lack of information on welfare traits in goats, especially in extensive production systems. Tethering is a common management practice in goat production in Africa (Mataveia et al., 2018), and while it serves to protect the animals from stock theft it also restricts their feeding and often leads to both malnutrition and dehydration.

## 4 Mitigating strategies

Genomic technology provides the opportunity to develop mitigating strategies to manage the unintended negative consequences of directional selection. Several DNA markers such as microsatellite markers have been used since the early 1990s for DNA-based parentage verification and identification of genetic defects. The completion of the full sequence of the reference bovine genome in 2009 (Matukumalli et al., 2009) and the subsequent discovery of single-nucleotide polymorphism (SNP) markers have provided numerous opportunities for studying genetic defects, developing commercial diagnostic tests and SNP-based parentage verification panels, as well as routine genotyping for genomic selection and management of genomic inbreeding.

### 4.1 Diagnostic testing

A large number of inherited disorders and familial traits in animals (Lenffer et al., 2006) have been catalogued in the Online Mendelian Inheritance in Animals (OMIA). This catalogue is continuously updated as new phenotypes are observed and confirmed as inherited disorders. The origins of most genetic defects remain uncertain. However, it is fair to assume that some were fixated within certain breeds due to linkage with economically important traits (thus as a consequence of selection for production). Some were disseminated throughout populations and breeds by the use of reproductive technologies such as AI and the overuse of some superior, high-impact sires. SNP genotyping is an efficient methodology to identify carriers of genetic disorders (Ciepłoch et al., 2017). The causative mutations associated with a large number of defects are known and has been included on commercially available SNP arrays, to be identified during routine genotyping.

Success stories of the eradication of genetic disorders using DNA-based diagnostic testing include neuropathic hydrocephalus found in Angus cattle populations (Teseling and Parnell, 2013) and cardiomyopathy and woolly hair coat syndrome (CWH) in Hereford cattle (Simpson et al., 2009). Several other genetic disorders affecting beef cattle, sheep and pigs can be identified using diagnostic tests. Some of these are available as either single gene-based tests or were added to commercial SNP arrays. Table 3 presents a summary of genetic defects available on the SNP arrays for cattle, sheep and pigs, which can be requested in routine genotyping.

**Table 3:** Summary of the number of genetic defects included on SNP arrays for various livestock species.

Species	SNP array	No.	Reference
		defects	
Beef	Beef GGP HD150K array	48	Illumina (2020, 2015a)
Dairy	Dairy GGP 50K array and dairy GGP HD150K array	18	Illumina (2020); Neogen Corporation (2020a)
Pigs	Porcine 60KSNP array	5	Illumina (2015b); Neogen Corporation (2020b)
Sheep	Ovine SNP50 beadchip	21	Neogen Corporation (2020c); https://SheepHapMap.org
			(last access: 3 September 2020); Synnov (2016)

Genetic defects can be considered as part of animal welfare as the birth of abnormal offspring should be prevented. Normal calving is regarded as a risk to welfare, due to the accompanying pain, stress and risk of dystocia (Ritter et al., 2019). Giving birth to an abnormal or stillborn calf is an unnecessary additional trauma for the female animal as well as a production loss for the farmer. Diagnostic testing can be used to eliminate genetic defects from populations by identifying phenotypically normal carrier animals (heterozygotes) and preventing their mating with similar carriers. Genetic disorders with significant economic impacts can therefore be removed from the breeding population and culled to eradicate the causative mutation completely.

### 4.2 Management of inbreeding levels

The adverse effects of inbreeding have been well documented across all livestock species (Weigel, 2001; Andersson, 2013). Incorrect and incomplete pedigree records however remain a main contributor to increased and unacceptable levels of inbreeding. One of the first and still one of the most important contributions of molecular genetics to livestock breeding was the development of parentage verification panels. These panels commonly consisted of a small number of microsatellite markers but are now included as routine genotyping panels in SNP arrays. Correct parentage is further crucial for accurately estimated genetic parameters (Visscher et al., 2002; Pollak, 2005), breeding values and rate of genetic progress (Van Eenennaam and Drake, 2012; Garritsen et al., 2015). In both the dairy and beef industries, breeders have the opportunity to use SNP-based parentage testing and confirm carrier animals with genetic defects as part of routine genotyping. These technologies are still primarily available to commercial farmers in developed countries and remain unaffordable for the majority of developing countries.

### 4.3 Genomic selection

Genomic selection (GS) is the selection of animals for breeding based on both their estimated breeding values and a direct genomic value, calculated from the combined effects of markers spread across the genome (Goddard and Hayes, 2009; Meuwissen et al., 2016). GS is most effective when applied to traits that are difficult to measure, expressed in only one gender or expressed late in life. This made GS an attractive technology for the dairy cattle industry, which was the first of the livestock industries to adopt the methodology.

The implementation of GS required training populations and phenotypes. Due to extensive use of AI in the dairy industry, they took the lead in implementation of GS. As indicated by Berry et al. (2014), larger training populations are required for lowly heritable traits. Studies have however proven the impact on the rate of genetic improvement in traits such as fertility, when genomic information was added in genetic evaluations (Garcia-Ruiz et al., 2016; Ma et al., 2018). McNeel et al. (2017) demonstrated the value of genomic information for prediction for a number of health and welfare traits, which included lameness and mastitis. Genomic information was added to the genetic evaluations to provide a standard transmitting ability (STA) for the animals. The authors however stressed the importance of available phenotypes for these traits related to health and welfare.

Genomic selection has also been applied in beef cattle worldwide where large numbers of beef cattle have been genotyped. Reliability of predictions improved with the use of genomic information and shows value, especially for the difficult to measure traits such as feed efficiency (Hayes et al., 2013). Limited GS has been used for the improvement of health and welfare traits in beef cattle. Despite the potential of genomic information for selection of welfare and health traits, accurate phenotypes remain a prerequisite for use of the methodology (Coffey, 2020) and are currently the main challenge for implementation.

The field of genomics has shown significant progress in the past decade, and genomic selection has become the method of choice to attain fast genetic progress. The benefits of this methodology are however limited to traits for which a large number of accurate phenotypes are available (Visser et al., 2020). Phenotyping is thus the limiting factor for improving reproductive and other welfare traits, such as clinical mastitis, claw lesions and hoof scoring. Countries with substantial phenotypes will reap the benefits of genomics, while in developing countries such as South Africa, welfare traits are not routinely recorded. Genomics will not improve animal welfare if proper farm animal management practices, animal recording and phenotyping are not in place. This must be supported by an increased consciousness for animal welfare by animal scientists, farmers, industry and government.

## 5 Conclusions

The selection emphasis on production traits has clearly resulted in a decrease in fitness traits including fertility, health and welfare of livestock. Selection for welfare and health traits is complex and most of the time not part of routine recording. It is necessary to find solutions to mitigate the unintended consequences of directional selection and to attain balanced breeding objectives that can be applied in sustainable breeding and efficient animal production. Genomics is one of the tools that can be used to select against genetic defects, control inbreeding and add genomic information for increased accuracy. This, however, should be reinforced by good management practices and an increased awareness of the importance of animal wellbeing.

## Data Availability

No data sets were used in this article.
